# Limited onward transmission potential of reassortment genotypes from chickens co-infected with H9N2 and H7N9 avian influenza viruses

**DOI:** 10.1080/22221751.2021.1996209

**Published:** 2021-11-03

**Authors:** Wen Su, Sin Fun Sia, Ka-Tim Choy, Yue Ji, Dongdong Chen, Eric Ho Yin Lau, Guanghua Fu, Yu Huang, Jinhua Liu, Malik Peiris, Juan Pu, Hui-Ling Yen

**Affiliations:** a School of Public Health, Li Ka Shing Faculty of Medicine, The University of Hong Kong, Hong Kong Special Administrative Region, People’s Republic of China; b Institute of Animal Husbandry and Veterinary Medicine, Fujian Academy of Agricultural Sciences, Fuzhou, People’s Republic of China; c Key Laboratory of Animal Epidemiology and Zoonosis, Ministry of Agriculture, College of Veterinary Medicine, China Agricultural University, Beijing, People’s Republic of China

**Keywords:** Co-infection, reassortment, H9N2, poultry, transmission chain

## Abstract

The segmented genome of influenza A virus has conferred significant evolutionary advantages to this virus through genetic reassortment, a mechanism that facilitates the rapid expansion of viral genetic diversity upon influenza co-infections. Therefore, co-infection of genetically diverse avian influenza viruses in poultry may pose a significant public health risk in generating novel reassortants with increased zoonotic potential. This study investigated the reassortment patterns of a Pearl River Delta-lineage avian influenza A(H7N9) virus and four genetically divergent avian influenza A(H9N2) viruses upon co-infection in embryonated chicken eggs and chickens. To characterize “within-host” and “between-host” genetic diversity, we further monitored the viral genotypes that were subsequently transmitted to contact chickens in serial transmission experiments. We observed that co-infection with A(H7N9) and A(H9N2) viruses may lead to the emergence of novel reassortant viruses *in ovo* and in chickens, albeit with different reassortment patterns. Novel reassortants detected in donor chickens co-infected with different combinations of the same A(H7N9) virus and different A(H9N2) viruses showed distinct onward transmission potential to contact chickens. Sequential transmission of novel reassortant viruses was only observed in one out of four co-infection combinations. Our results demonstrated different patterns by which influenza viruses may acquire genetic diversity through co-infection *in ovo*, *in vivo*, and under sequential transmission conditions.

## Introduction

Avian influenza viruses (AIVs) are the primordial source of influenza viruses that have established themselves in humans and other animal species [1]. Through adaptive mutations and genetic reassortments, AIVs may continue to drive the generation of the next influenza pandemic [2]. Live poultry markets, where birds infected with genetically diverse AIVs congregate are known to facilitate co-infections and the emergence of novel AIVs with increased zoonotic potential [3]. However, the onward transmission potential of novel reassortant genotypes generated after co-infections is currently unknown.

Recent studies have provided valuable insights into the mechanisms driving the genetic reassortment of influenza A viruses [4]. Using two homologous influenza A(H3N2) viruses that differ by 2–6 synonymous mutations in each segment, previous studies have shown that homologous reassortment is highly efficient, although the co-infection conditions may determine the reassortment frequency of the viral progeny [5–7]. For example, in vitro co-infection at a high multiplicity of infection (MOI) of 10 plaque-forming units (PFU)/cell supported a high level of reassortment, with an average reassortment frequency of 88.4%; however, the reassortment frequency dropped to 9.5% at an MOI of 0.01 [5]. In comparison, heterologous reassortments are less efficient in generating genetically diverse viral progeny due to vRNA-vRNA or protein-protein incompatibilities [4]. For example, the packaging signals restricted genetic shift of *haemagglutinin* (*HA*) segments from H5N2 or H7N9 AIVs to human influenza A(H3N2) viruses [8,9]. Protein incompatibility in forming a functional heterotrimer between polymerase acidic protein (PA) of equine influenza A(H7N7) virus and polymerase basic protein 2 (PB2) and 1 (PB1) of A(H3N2) virus limited genetic reassortment between the two heterologous viruses [10]. Although guinea pigs co-infected with two homologous viruses could transmit reassortants to co-housed contact guinea pigs [7], there have been limited studies focusing on the co-infection of heterologous viruses and the onward transmission potential of reassortants in animal models.

Avian influenza A(H9N2) viruses are enzootic among terrestrial poultry species in Asian, Middle Eastern, and African countries [11]. A(H9N2) viruses have phylogenetically established at least three lineages, the G1-like lineage, the Y280-like lineage (also known as BJ94 or G9 lineages) and the Y439 lineage [12]. Human infections by A(H9N2) viruses have been sporadically reported mainly from both G1-like and Y280-like lineages [11], with most infections leading to mild clinical signs in children [13]. In addition to directly causing new infections in humans [14,15], A(H9N2) viruses have contributed to the internal genes of multiple avian influenza strains that have caused lethal human infections in China [16]. The internal genes of the G1-like lineage A(H9N2) viruses are genetically related to those of the 1997 A(H5N1) viruses [17]. Since 2010, genotype 57 (G57), which evolved from the Y280-like lineage A(H9N2) viruses, has replaced other genotypes and has gained dominance in chickens [18]. The G57 A(H9N2) viruses subsequently donated all six internal gene segments to A(H7N9) viruses that emerged in 2013 [18,19]. A(H9N2) viruses also provided internal genes for the emergence of A(H5N6), A(H10N8) and A(H10N3) viruses that have zoonotic potential [20–22].

Among all known AIVs that have caused human zoonotic infections, the 2013 A(H7N9) virus is of most concern as the virus has caused 1568 confirmed human infections including 616 fatal cases [15]. The low pathogenic A(H7N9) viruses were generated by multiple reassortments, comprising genes from AIVs detected in ducks, migratory birds and chickens [23]. They have evolved into two lineages (Yangzte River Delta lineage and Pearl River Delta lineage) with multiple transient genotypes [24–26]. In late 2016, highly pathogenic A(H7N9) virus with insertion of multiple basic amino acid residues at the HA cleavage site emerged from the Yangtze River Delta lineage [27]. Although there was extensive genetic diversity present among A(H7N9) viruses, the virus predominantly obtained internal genes from A(H9N2) viruses that belongs to G57, which has been continuously evolving in chickens [28,29].

A(H9N2) viruses were found co-circulating in poultry with A(H7N9) viruses in China [30,31]. While A(H7N9) and A(H9N2) viruses share homologous internal genes, the reassortment patterns of different A(H9N2) viruses co-infected with the A(H7N9) viru*s in vitro* and *in vivo* have not been evaluated experimentally. Here, we aimed to characterize viral progenies generated after co-infection of A(H7N9) and A(H9N2) viruses of different genetic lineages *in ovo* and in White Leghorn chickens. We also monitored viral genotypes that were subsequently transmitted to the chickens in serial transmission experiments. Our results have implications for the evolution and transmission dynamics of AIV in avian hosts where co-infections might occur.

## Materials and methods

### Ethics, biosafety, and biosecurity statement

Risk assessment of co-infection protocols was conducted by the Office of Biological Safety at the University of Hong Kong (HKU). Chicken experiments were approved by the Committee on the Use of Live Animals in Teaching and Research at the HKU (#4532-17). All experiments using A(H7N9) virus were performed in a biosafety level 3 facility with restricted access following approved standard operating procedures at HKU.

### Cells

Madin-Darby canine kidney (MDCK) cells were maintained in modified Eagle’s medium (MEM) supplemented with 10% fetal calf serum (FCS), 1% penicillin-streptomycin (P/S), 1% vitamin, and 25 mM HEPES (Gibco). Human embryonic kidney (293T) cells were maintained in Opti-MEM supplemented with 5% FCS and 1% P/S.

### Viruses

A/silkie Chicken/Hong Kong/1772/2014(H7N9) (designated as HK1772; GISAID accession # EPI_ISL_4882548) represents the Pearl River Delta lineage A(H7N9) viruses. The transmissibility of HK1772 in chickens had been described well previously [32]. A/chicken/Beijing/16/2013(H9N2) (designated as BJ16; GISAID accession # EPI_ISL_3144854) virus is a representative strain of G57 H9N2 viruses that co-circulated with the A(H7N9) viruses [18]. Other A(H9N2) viruses included in this study serve as comparators to BJ16. Specifically, A/chicken/Zhejiang/HJ/2007 (designated as HJ; GISAID accession # EPI_ISL_3144489) is an early G57 H9N2 virus [18], while A/silkie chicken/Hong Kong/YU335/2007 (designated as YU335; GISAID accession # EPI_ISL_3144467) belongs to G44 that circulated prior to the G57 viruses [33]. A/quail/Hong Kong/G1/1997 (designated as G1; GISAID accession # EPI_ISL_3144464)-like viruses has been established in the Middle East countries and was also included in the study [34]. HK1772, G1 and YU335 viruses were stored in our lab at HKU. HJ and BJ16 viruses were generated by plasmid-based reverse genetics [35]. Viruses were propagated one time in 10-day old specific-pathogen-free (SPF) embryonated chicken eggs (JINAN SPAFAS Poultry Co., Ltd, China) and confirmed by next-generation sequencing (NGS). Viral titres were determined by plaque assay on MDCK cells or in eggs at 50% egg infective dose (EID_50_) [36].

### Infection in embryonated chicken eggs

Three 10-day old embryonated chicken eggs were infected with 10^4^ PFU or 10^6^ PFU of virus alone or a mixture of the A(H7N9) virus and one of the four A(H9N2) viruses at a 1:1 ratio with 10^4^ PFU of each virus in a volume of 0.1 mL. After injection, eggs were incubated at 35°C and monitored routinely. Allantoic fluid was harvested at 1, 8, 16 or 48 hour post-infection (hpi) and was stored at -80°C. Viral titres were determined by plaque assay on MDCK cells. Co-infecting samples were further analysed by genotyping and NGS. Sequencing data are available in the NCBI BioProject PRJNA768344.

### Plaque purification

Viruses were purified using a modified plaque assay. Confluent MDCK cells in 100 mm dishes were infected with original samples and incubated at 37°C. At 70 hpi, plaques were visualized by 1% agarose containing 0.5 mg/mL MTT (Sigma-Aldrich). Individual plaques were picked randomly using 1 mL barrier tips. RNA was extracted from the agar plug using the QIAamp Viral RNA Mini Kit (Qiagen) according to the manufacturer’s instructions.

### Determination of virus genotypes by high resolution melting analysis

A multiplex real-time RT-PCR was employed to simultaneously type *H7/H9* and *N2/N9* gene segments based on a multiplex probe combination on a ViiA 7 Real-Time PCR system (Thermo Fisher Scientific). Experiments were performed in 25 µL reaction mixtures with 5 µL of viral RNA and AgPath-ID One-Step RT-PCR reagents (Life Technology), in duplicate, according to the manufacturer’s instructions. High resolution melting (HRM) analysis was performed to differentiate six internal genes of the A(H7N9) virus from those of co-infected A(H9N2) viruses using LightCycler Gene Scanning Software according to the manufacturer’s instructions. Each internal gene segment was identified in 10 µL reaction mixtures containing 1 µL of the cDNA and HRM master mix (Roche) in 384-well plates, in duplicate, on the LightCycler 480 II instrument (Roche). The HRM curve was analyzed using the Light Cycler Software (Version 1.5). If any segment from a given plaque could not be determined, the plaque was not included in the reassortment analysis. Sequences of primers and TaqMan probes used in the study were listed in sTable 1. They were synthesized by Integrated DNA Technologies (IDT, Singapore).

### Co-infection and transmission in chickens

White Leghorn chickens were hatched from SPF eggs and raised in a clean environment. Thirty-six 5-week-old chickens were randomly separated into four groups. For every group, three chickens (designated as donors) were inoculated intranasally with a mixture of the H7N9 virus and one H9N2 strain (10^6^ EID_50_ per virus) in 500 µL phosphate-buffered saline. At 1-day post-infection (dpi), three donors were moved and housed with three naïve contacts (designated as 1st contacts) that were separately housed in different cages. At 3 dpi, three 1st contacts were co-housed with three naïve chickens (designated as 2nd contact) in other three cages. At 5 dpi, all chickens were single-housed until the end of the experiment (17 dpi). Oropharyngeal swabs and cloacal swabs were collected from donors at 1, 2 and 3 dpi. Donors were euthanized at 3 dpi. Oropharyngeal swabs and cloacal swabs were collected from 1st contacts at 2, 3, 4, 5, 7, 9, 11 and 13 dpi and 2nd contacts at 4, 5, 7, 9, 11 and 13 dpi. All contacts were euthanized at 17 dpi. This experimental setting allowed assessing three independent primary transmission events (from donors to 1st contacts) and three secondary transmission events (from 1st contacts to 2nd contacts) under each combination of H7N9 and H9N2 co-infection. Totally, this chicken experiment contained 12 serially independent chicken transmission chains.

Influenza virus *M* gene copy numbers in the swabs were determined by quantitative real-time RT-PCR using the ViiA 7 System. Plaques isolated from original oropharyngeal swabs of donors at 1 and 3 dpi, 1st contacts at 3 dpi (2 days post-exposure (dpe)) and 2nd contacts at 5 dpi (2 dpe) were further genotyped. Oropharyngeal swabs of donors at 3 dpi were analyzed by NGS. Sequencing data are available in the NCBI BioProject PRJNA769384. Pre- and post-sera were collected at -1 and 17 dpi from chickens for the detection of anti-HA antibody using the OIE’s haemagglutination inhibition assay (HI) [37] or anti-influenza A NP antibody using an ID Screen influenza A virus antibody competition enzyme-linked immunosorbent assay (ELISA) kit (ID.vet, France) according to the manufacturer’s instructions.

### Statistical analysis

Data were analyzed using GraphPad Prism version 8.4.1 (GraphPad Software) and R version 4.0.4 (R Development Core Team). Wilcoxon matched-pair signed-rank test was used to compare the difference of viral loads between oropharyngeal swabs and cloacal swabs. Kruskal-Wallis test was used to compare the genotype diversity index (GDI), and area under the curve (AUC) of viral loads of four groups followed by Dunn’s multiple comparisons tests for pairwise comparisons. Fisher's exact test with Bonferroni correction was performed to identify the correlation of genetic reassortment in eggs with donor chickens. Difference was considered statistically significant at *P* < .05.

## Results

### Diverse genotypes were detected after co-infection of A(H9N2) and A(H7N9) viruses in ovo

**Figure 1.**
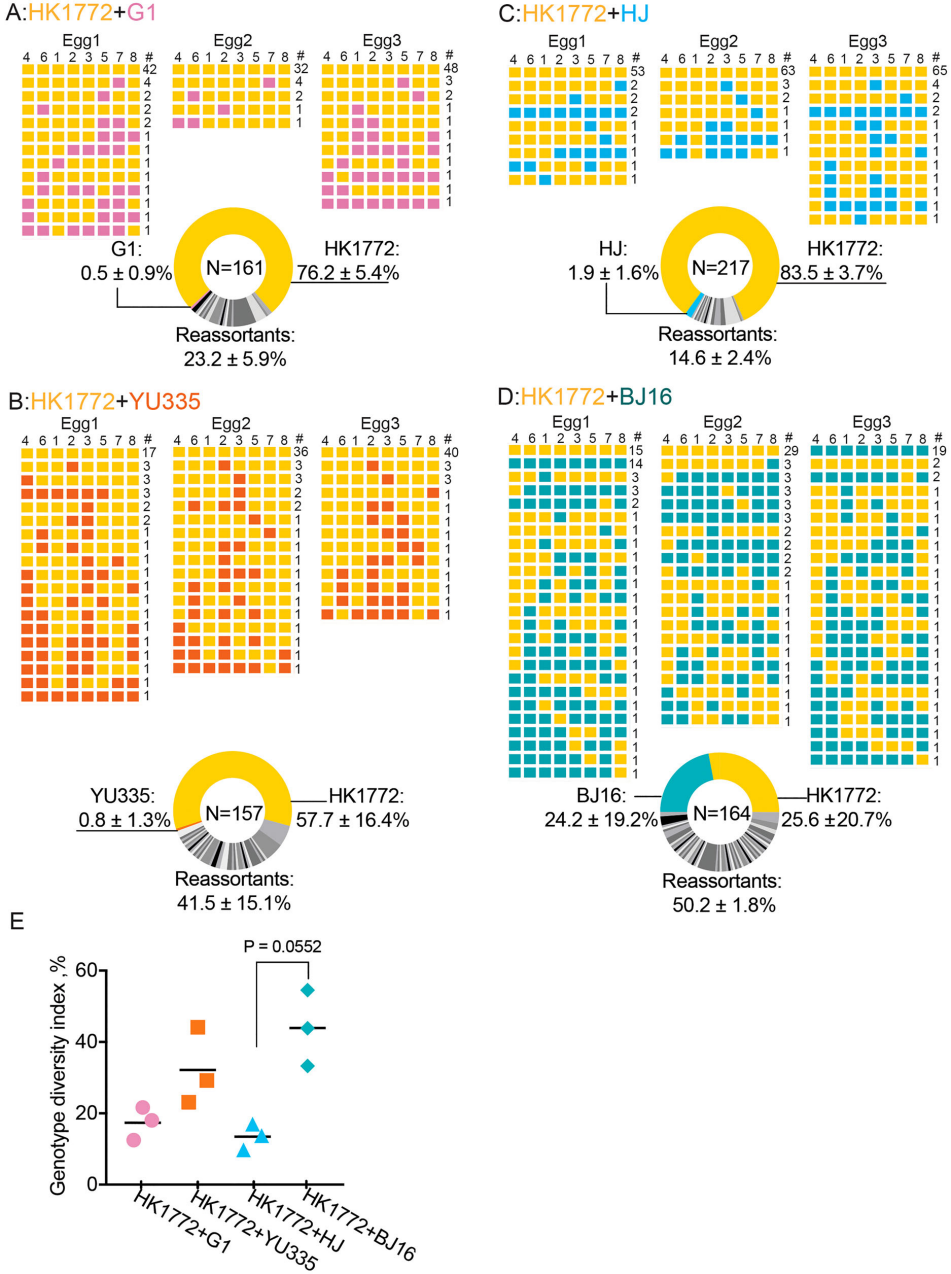
Reassortment between A(H7N9) and genetically divergent A(H9N2) viruses might occur in eggs. The parental A(H7N9) virus HK1772 is shown in orange. The parental A(H9N2) viruses: G1 (a), YU335 (b), HJ (c), and BJ16 (d) are shown in purple, red, blue, and cyan, respectively. HA(4), NA(6), PB2(1), PB1(2), PA(3), NP(5), M(7), and NS(8) of plaques are shown. # represents the number of plaques with the same genotype. In the pie charts, N indicates the total number of plaques examined in this combination. The mean ± standard deviation (SD) percent of genotype frequency is shown. New genotypes are shown in different shades of grey. (e) Genotype diversity index is the number of different genotypes divided by the total number of plaques screened in the egg. Statistical analyses were performed by Kruskal-Wallis test followed by Dunn’s multiple comparison test. The minimum P values are shown.

To investigate progenies generated after co-infection of A(H7N9) and A(H9N2) viruses, we first characterized the A(H7N9) virus HK1772 and four genetically divergent A(H9N2) viruses *in vitro.* The four A(H9N2) viruses replicated well *in ovo* (sFigure 1(a)), with a higher increment at the dose of 10^4^ PFU than 10^6^ PFU at 16 hpi (*P* = .029, Mann-Whitney test) (sFigure 1(b)). They including the A(H7N9) strain HK1772 formed visible plaques in MDCK cells, although the plaque sizes were not identical (sFigure 1(c)). Co-infection was performed by co-infecting eggs in triplicate with 10^4^ PFU/virus of H7N9 and H9N2 viruses, and allantoic fluid was harvested at 16 hpi (peak titres) for viral genotype analysis. Plaques were isolated from three eggs co-infected with HK1772+G1 (*N* = 161) (Figure 1(a)), HK1772+YU335 (*N* = 157) (Figure 1(b)), HK1772+HJ (*N* = 217) (Figure 1(c)), or HK1772+BJ16 (*N* = 164) (Figure 1(d)). Reassortants were detected at 23.2 ± 5.9%, 41.5 ± 15.1%, 14.6 ± 2.4%, and 50.2 ± 1.8%, from three eggs co-infected with HK1772+G1 (Figure 1(a)), HK1772+YU335 (Figure 1(b)), HK1772+HJ (Figure 1(c)), and HK1772+BJ16 (Figure 1(d)), respectively. The parental HK1772 virus was most prevalent (>50%) in eggs co-infected with HK1772+G1 (76.2%), HK1772+YU335 (57.7%), and HK1772+HJ (83.5%) (Figure 1(a–c)). NGS analyses confirmed that gene segments of the HK1772 virus were dominant in eggs co-infected with HK1772+G1, HK1772+YU335, and HK1772+HJ (sFigures 2–5).

Next, we used genotype diversity index (GDI = the number of genotypes divided by the number of plaques identified) to quantify the extent of reassortment. The GDI significantly varied among four combinations (Kruskal-Wallis test, *P* = .0039). Specially, the GDI of HK1772+BJ and HK1772+HJ combinations differed the most, but insignificant (*P* = .055, Dunn’s post-hoc test)(Figure 1(e)). These results suggest that the A(H7N9) virus might possess a competitive advantage over G1, YU335, HJ, but not BJ16 virus *in ovo*. Upon co-infection, these A(H9N2) viruses have different capacities to generate novel reassortant genotypes with the A(H7N9) virus *in ovo*, with novel reassortant genotypes detected at mean frequencies of 14.6 to 50.2%.

### Transmission dynamics of chickens co-infected with A(H7N9) and A(H9N2) viruses

**Figure 2.**
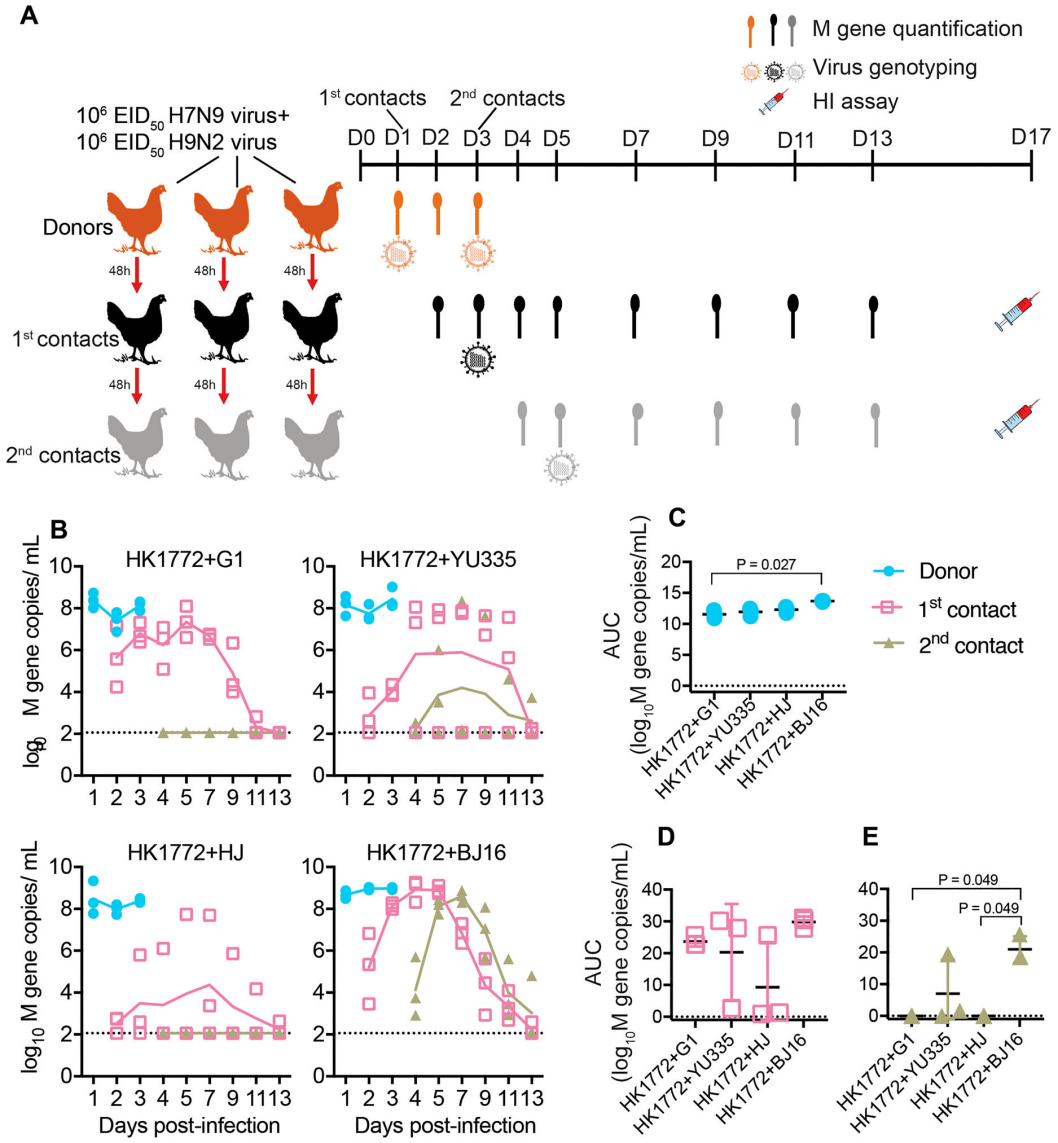
The onward transmission of reassortants in chickens co-infected with A(H7N9) and A(H9N2) viruses varied with strains in chickens. (a) Experimental scheme. Three chickens (designated as donors) were inoculated intranasally with a mixture of A(H7N9) and A(H9N2) viruses. At 1-day post-infection (D1), three donors were housed with three naïve contacts (designated as 1st contacts) in three cages. At D3, three 1st contacts were co-housed with three naïve chickens (designated as 2nd contact) in three cages. At D5, all chickens were single-housed. Oropharyngeal and cloacal swabs and sera were collected at these indicated time points; HI, hemagglutinin inhibition; (b) Viral loads detected in oropharyngeal swabs. The lines represent the average viral M gene copies of three chickens (dots). Black horizontal dashed lines represent the limit of detection. (c, d, and e) The area under the curve (AUC) of viral loads in oropharyngeal swabs. Statistical analyses were performed by Kruskal-Wallis test followed by Dunn’s multiple comparison test. The *P* < .05 values are shown.

To characterize “within-host” and “between-host” genotype diversity, we performed sequential transmission experiments in chickens after co-infection of A(H7N9) and A(H9N2) viruses (Figure 2(a)). Four combinations replicated well in donor chickens, but with various onward transmission potential (Figure 2(b)). None of the chickens showed apparent clinical signs during the course of experiment; NGS analyses on oropharyngeal swabs of donors did not detect insertion at the HA cleavage site suggesting no detection of highly pathogenic A(H7N9) virus. The AUC was calculated to approximate viral loads. Higher viral loads were detected in the oropharyngeal swabs than in the cloacal swabs of all infected chickens (*P* = .002, Wilcoxon matched-pair signed-rank test) (Figure 2(b) and sFigure 6). Viral loads detected in the oropharyngeal swabs of donors varied significantly among four combinations (*P* = .027, Kruskal-Wallis test), donors co-infected with HK1772+BJ16 of which had higher viral loads compared to those co-infected with HK1772+G1 (*P* = .027, Dunn’s post hoc test) (Figure 2(c)).

Onward transmission from co-infected donors to 1st contacts was observed for all four H7N9+H9N2 combinations, but at different efficiencies (Figure 2(b)). The AUCs calculated from the oropharyngeal swabs of 1st contacts exposed to donors co-infected with HK1772+G1 (3/3 tested positive for M gene and seroconverted), HK1772+YU335 (2/3 tested positive for M gene and seroconverted), HK1772+HJ (2/3 tested positive for M gene and 1/3 seroconverted), or HK1772+BJ16 (3/3 tested positive for M gene and seroconverted) were not significantly different (Figure 2(d) and sTable 2). In contrast, transmission from the 1st contact to the 2nd contacts was limited, with 1/3 of the 2nd contacts infected in the HK1772+YU335 group and 3/3 of the 2nd contacts infected in the HK1772+BJ16 group. Viral loads detected in the oropharyngeal swabs of 2nd contacts varied significantly among four combinations (*P* = .032, Kruskal-Wallis test), and the 2nd contacts in the HK1772+BJ16 group shed the highest number of viruses (*P* = .049, Dunn’s post hoc test) (Figure 2(e)). Overall, four A(H7N9) and A(H9N2) combinations replicated well in donor chickens, with HK1772+BJ16 combinations showing the best replication capacity and onward transmission potential.

### Virus genotypes detected in donor chickens co-infected with A(H7N9) and A(H9N2) viruses

**Figure 3.**
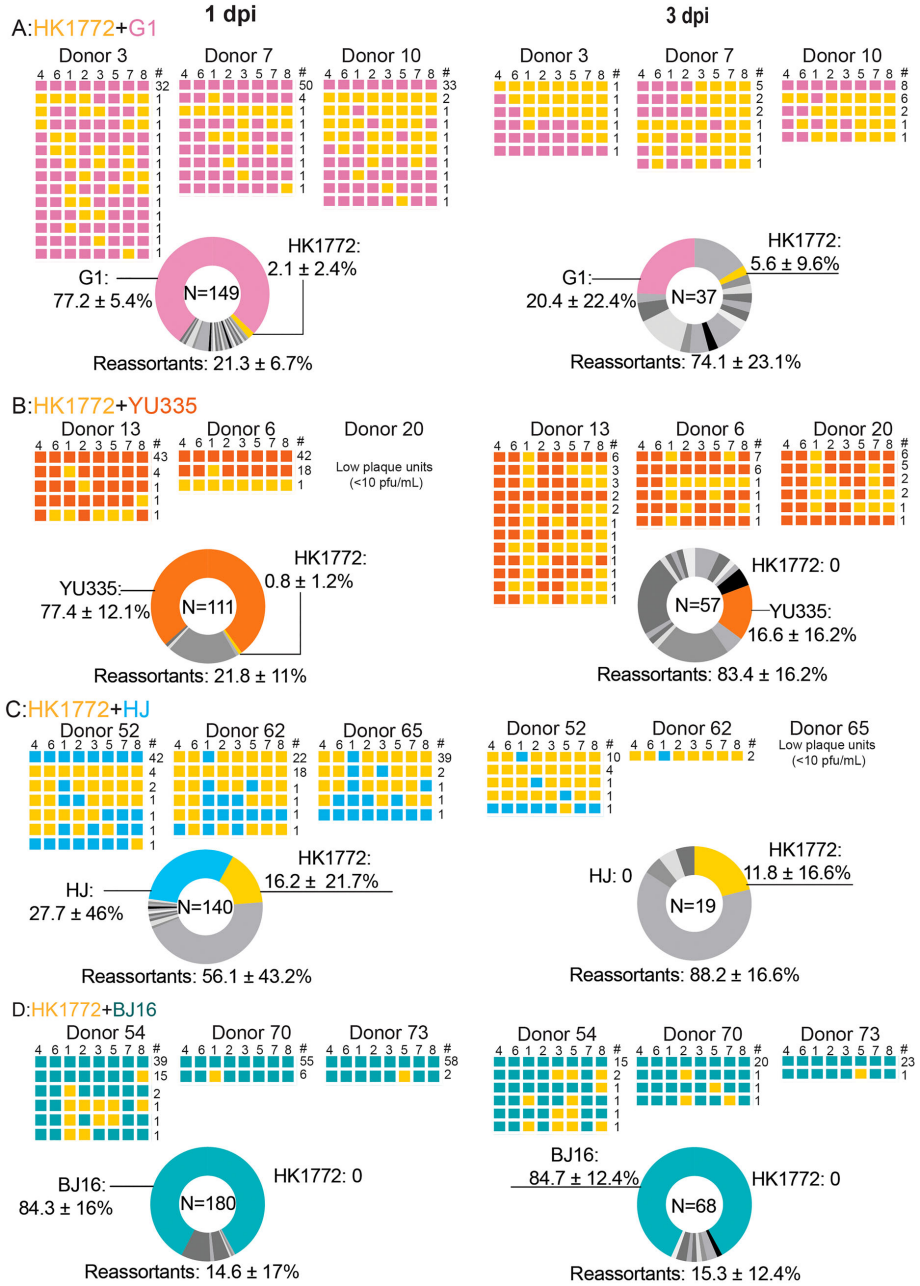
Robust reassortment could be detected in chickens co-infected with A(H7N9) and different A(H9N2) viruses. The parental A(H7N9) virus HK1772 is shown in orange. The parental A(H9N2) viruses: G1 (a), YU335 (b), HJ (c), and BJ16 (d) are shown in purple, red, blue, and cyan, respectively. HA(4), NA(6), PB2(1), PB1(2), PA(3), NP(5), M(7), and NS(8) of individual plaques are shown. # represents the number of plaques with the same genotype. In the pie charts, N indicates the total number of plaques examined in this combination. The mean ± SD percent of genotype frequency is shown. New genotypes are shown in different shades of grey.

To determine virus genotypes in donors co-infected with HK1772+G1 (Figure 3(a)), HK1772+YU335 (Figure 3(b)), HK1772+HJ (Figure 3(c)), and HK1772+BJ16 (Figure 3(d)), plaques in the oropharyngeal swabs sampled from three donors at 1 and 3 dpi were genotyped. At 1 dpi, the parental A(H9N2) viruses were the most prevalent (> 50%) from donors co-infected with HK1772+G1 (77.2%), HK1772+YU335 (77.4%), and HK1772+BJ16 (84.3%). At 3 dpi, reassortants were the most prevalent in donors co-infected with HK1772+G1 (74.1%), HK1772+YU335 (83.4%), and HK1772+HJ (88.2%). Interestingly, in donors co-infected with HK1772+BJ16, the parental BJ16 virus remained the dominant genotype at 1 and 3 dpi (Figure 3(d)), suggesting that BJ16 might possess a growth advantage over HK1772 in chickens.

Taken together, these results show that novel reassortants may be generated in chickens co-infected with A(H7N9) and A(H9N2) viruses, albeit at different efficiencies. Increased genetic diversity, as indicated by the higher detection frequencies for reassortant viruses, was observed in co-infected donors from 1 to 3 dpi (Figure 3). Interestingly, the frequencies of H7N9, H9N2, and reassortants detected *in ovo* and in chickens were not comparable (*P* < .001, Fisher’s exact test) (sTable 3), suggesting that in addition to virus-virus compatibility, differences in virus-host interactions may affect the efficiency of generating genetically diverse viral progeny after co-infections *in vivo*.

### Pairwise analysis showed distinct reassortment patterns after co-infection of A(H7N9) and A(H9N2) viruses *in ovo* and in chickens

**Figure 4.**
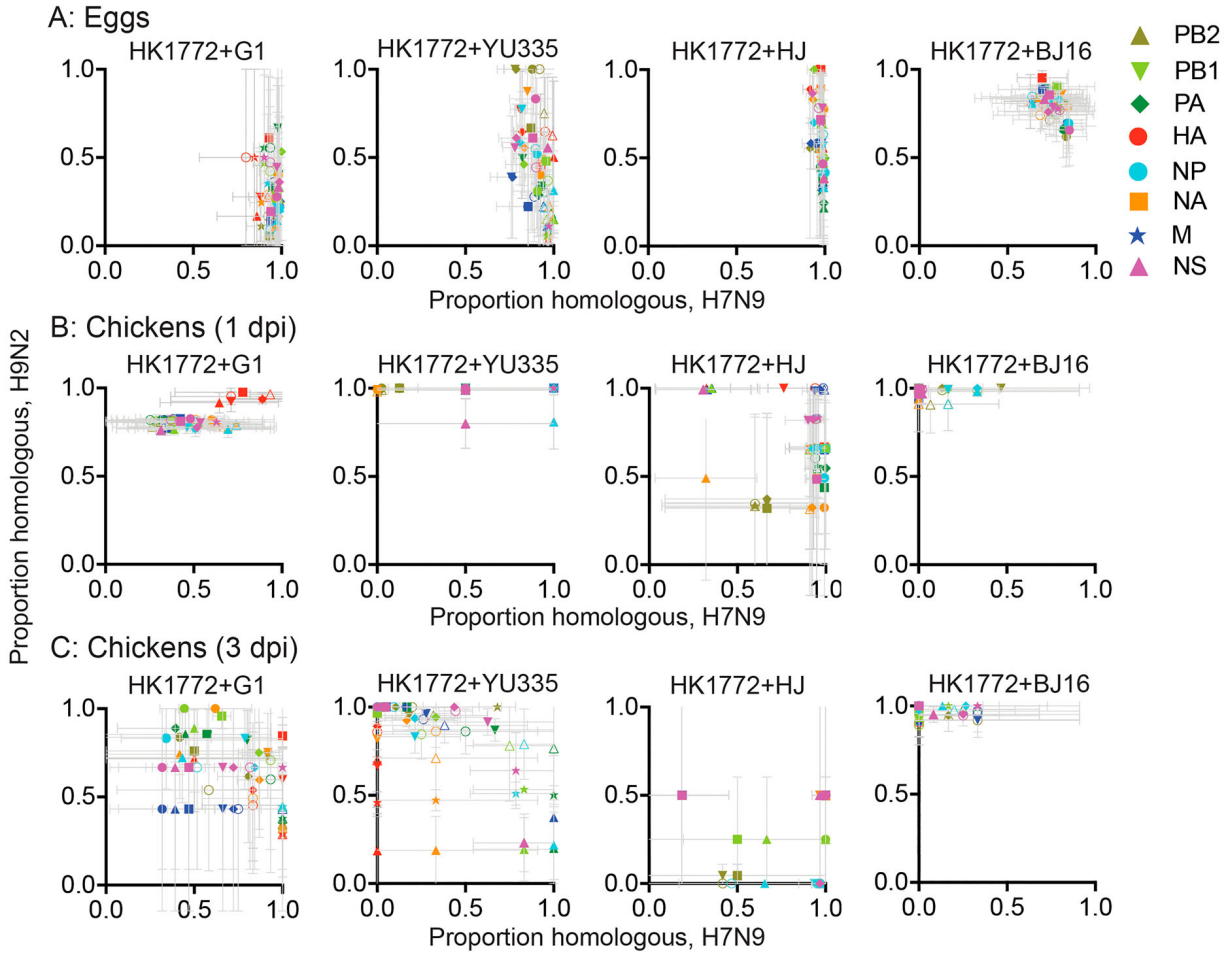
Pairwise comparison of genotyped segments showed various reassortment preferences between A(H7N9) and A(H9N2) viruses in eggs (a), and in oropharyngeal swabs collected at 1 dpi (b) and 3 dpi (c). Segments are shown with different symbols and colours. The means ± SD of homologous frequency from three independent co-infections are shown.

We further employed a pairwise quantified method [38] to evaluate whether a given segment randomly reassorts between two co-infected viruses *in ovo* and in chickens. When the proportion of homologous combinations falls in the centre of the graph, at 0.5 on each axis, it suggests that the two segments freely exchange. *In ovo*, data points of HK1772+G1, HK1772+YU335, and HK1772+HJ co-infections were shifted to the right of the X-axis, while the Y-axis data points were more evenly scattered between 0 and 1, suggesting a bias in maintaining homologous A(H7N9) gene segments in viral progenies (Figure 4(a)). This is consistent with the observation that the parental A(H7N9) virus HK1772 was detected (>50%) upon co-infection with G1, YU335, HJ, but not with BJ16 A(H9N2) viruses *in ovo* (Figure 1).

In chickens co-infected with HK1772+G1, HK1772+YU335, and HK1772+BJ16 at 1 dpi, Y-axis data points were shifted towards 1, suggesting a bias in maintaining homologous A(H9N2) gene segments in viral progenies (Figure 4(b)). This is consistent with the observation that the parental A(H9N2) viruses, G1, YU335, and BJ16, were detected (>50%) upon co-infection with HK1772 in chickens at 1 dpi (Figure 3). At 3 dpi, data points obtained from HK1772+G1 and HK1772+YU335 co-infected chickens were dispersed over a wider range, indicating an expansion in the genetic diversity of viral progeny as infection progressed over time (Figure 4(c)). In contrast, in chickens co-infected with HK1772+BJ16, the Y-axis data points have consistently shifted towards 1 at 1 and 3 dpi (Figure 4(b, c)), suggesting a continuous bias in maintaining homologous BJ16 gene segments in viral progenies and supporting the dominance of BJ16 in co-infected chickens.

### Novel reassortant viruses generated in co-infected donors demonstrated distinct onward transmission potential to contact chickens

**Figure 5.**
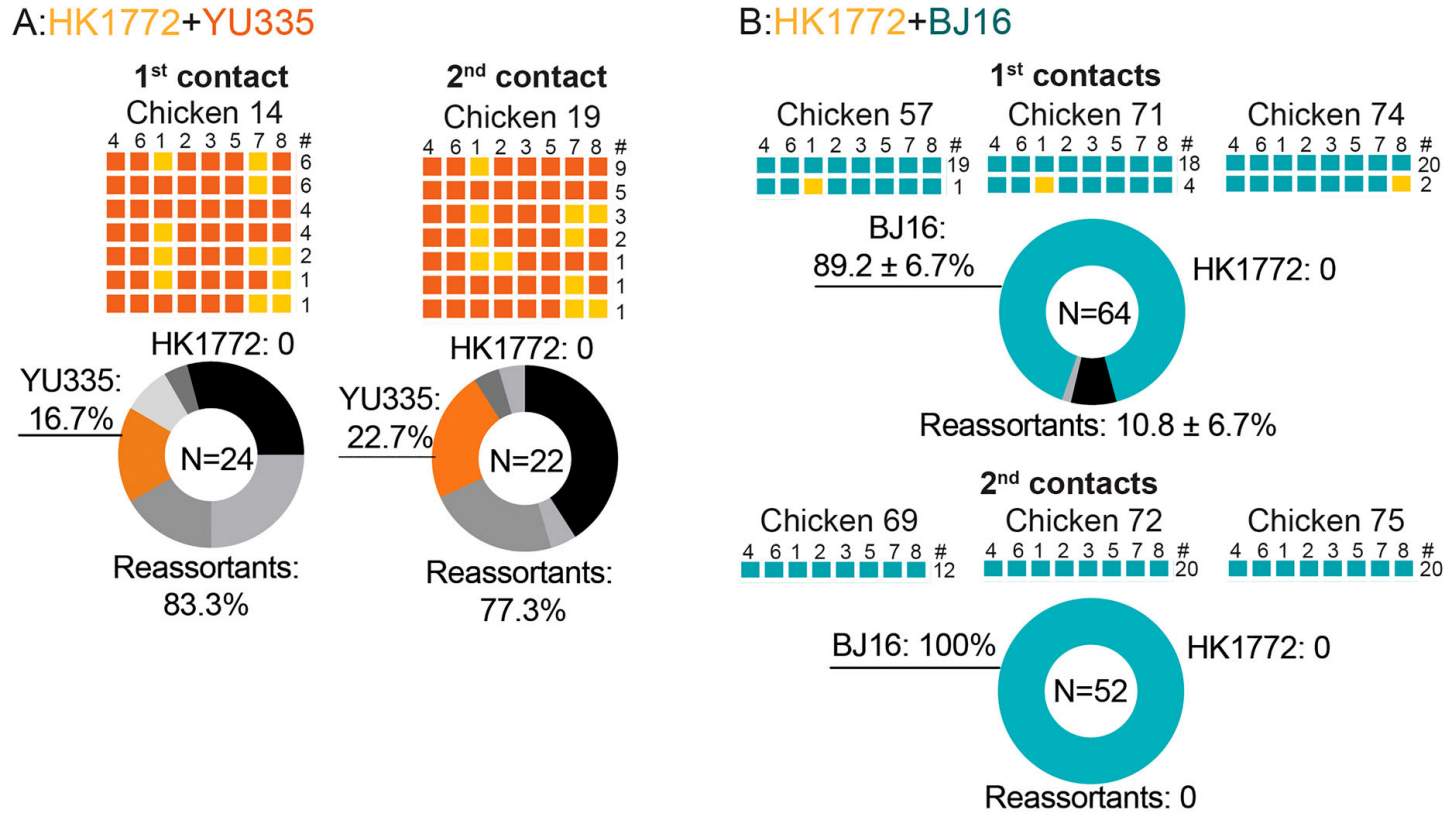
Viruses were transmitted to contact chickens. The parental A(H7N9) virus HK1772 is shown in orange. The parental A(H9N2) viruses :YU335 (a), and BJ16 (b) are shown in red and cyan, respectively. HA(4), NA(6), PB2(1), PB1(2), PA(3), NP(5), M(7), and NS(8) of individual plaques are shown. # represents the number of plaques with the same genotype. In the pie charts, N indicates the total number of plaques. The mean ± SD percent of genotype frequency is shown. New genotypes are shown in different shades of grey.

Since successful serial transmission was observed from the contacts in the HK1772+YU335 and HK1772+BJ16 groups, we genotyped the oropharyngeal swabs collected from these infected contacts (Figure 2(b)). In the HK1772+YU335 group, seven genotypes were detected from 24 plaques isolated from the 1st contact, and reassortants were the most prevalent 83.3% (Figure 5(a)), suggesting the co-transmission potential of the reassortant viruses. Onward transmission was only detected in 1/3 of the 2nd contacts. Among the seven genotypes detected in the infected 2nd contacts, six genotypes were identical to those detected in the 1st contact (Figure 5(a)). The GDI detected from the donor at 3 dpi (52.2%), the 1st contact (29.2%), and the 2nd contact (31.8%) were similar (Table 1), demonstrating the onward transmission potential of the novel reassortants.

**Table 1.** Genotype diversity index (%) detected in chickens. Genotype diversity index was determined by plaques isolated from oropharyngeal swabs of donors at 1 and 3 days post-infection (dpi), 1st contacts at 3 dpi and 2nd contacts at 5 dpi.

Virus combination	Donors		1st contacts	2nd contacts
	1 dpi	3 dpi		
HK1772+G1				
1	31.1%	100.0%	0.0%	0.0%
2	14.8%	53.8%	0.0%	0.0%
3	23.3%	27.8%	0.0%	0.0%
HK1772+YU335				
1	10.0%	52.2%	29.2%	31.8%
2	4.9%	35.3%	4.2%	0.0%
3	0.0%	35.3%	0.0%	0.0%
HK1772+HJ				
1	13.5%	29.4%	0.0%	0.0%
2	13.6%	100.0%	0.0%	0.0%
3	11.4%	0.0%	0.0%	0.0%
HK1772+BJ16				
1	10.2%	28.6%	10.0%	8.3%
2	3.3%	17.4%	9.1%	5.0%
3	3.3%	8.3%	9.1%	5.0%

In the HK1772+BJ16 group, as BJ16 was the dominant genotype in co-infected donors (Figure 3(d)), it was also the predominant genotype detected in the 1st contacts (89.2%) and 2nd contacts (100%) (Figure 5(b)). The total GDI detected from three donors at 3 dpi (13.2%), three 1st contacts (4.7%) and three 2nd contacts (1.9%) steadily declined (Table 1).

Taken together, we observed different transmission dynamics in chickens following co-infections with HK1772+YU335 and HK1772+BJ16 viruses. In the HK1772+YU335 group, novel reassortants were passed onwards from co-infected donors to the 1st and 2nd contacts, demonstrating the capacity of passing on genetically diverse variants after co-infections. In contrast, a dominant genotype (BJ16) was transmitted from donors to contact chickens in the HK1772+BJ16 group.

## Discussion

In this study, we compared the genetic diversity of viral progeny generated *in ovo* and *in vivo* upon co-infection with four combinations of A(H7N9) and A(H9N2) viruses. We further evaluated the onward transmission potential of novel reassortants in chickens. Despite of detecting multiple novel reassortants in donors co-infected with four combinations of A(H7N9) and A(H9N2) viruses, most of the novel reassortants were not detected in contact chickens after exposure. Onward transmission of novel reassortants from co-infected donors to the 1st and the 2nd contacts was only observed in the HK1772+YU335 group. Furthermore, among multiple novel reassortants detected in donors co-infected with HK1772+BJ16, only the parental BJ16 virus was transmitted to the 1st and 2nd contacts. Taken together, these findings demonstrate limited onward transmission potential of novel reassortants generated in chickens co-infected with A(H7N9) and A(H9N2) viruses.

We observed that co-infection with different A(H9N2) and the same A(H7N9) viruses may lead to the emergence of reassortants at different efficiency and with dissimilar patterns. Reassortment efficiency was strongly associated with timing, dose and spatial distribution of co-infection [6,7,39]. In the study, we simultaneously infected eggs and chickens with a high dose of A(H7N9) and A(H9N2) viruses. Cumulative evidences have indicated the critical roles of RNA-based intersegmental interactions in genetic reassortment of influenza A virus [40,41]. Physiological bottlenecks *in vivo* are narrower than that *in ovo*, possibly limiting genetic exchange of influenza viruses by decreasing the number of viruses that can attach to the cells to initiate co-infection [42,43]. Furthermore, differential tissue tropism may also affect reassortment frequency [44,45], as we have observed the dominance of A(H7N9) virus *in ovo* and the dominance of A(H9N2) viruses in donor chickens at 1 dpi. It remains to be studied if the A(H7N9) or A(H9N2) viruses possess superior replication fitness in eggs and chickens.

Our results showed that co-infections of A(H7N9) and different A(H9N2) viruses could support generation of novel genotypes *in ovo* and *in vivo*. Interestingly, most of novel reassortant genotypes detected in the co-infected donors were less likely to be transmitted onwards to contact chickens, with the exception of novel reassortant genotypes generated in donors co-infected with the H7N9 virus and YU335 virus. This result coincides with the surveillance findings that multiple H7N9 genotypes were transient and only a few genotypes have sustained more than two waves of A(H7N9) epidemics in 2013–2017 [24–26]. The determinants contributed to increased onward transmission potential of specific reassortant genotypes remain to be studied.

Our study has several limitations. First, genetic analyses may be biased towards reassortants that formed visible plaques and, therefore overlook progenies that didn’t form plaques. Second, A(H7N9) viruses have two main genetic lineages in China: the Pearl River Delta lineage and the Yangtze River Delta lineage [24]. The A(H7N9) strain HK1772 belongs to the Pearl River Delta lineage. Thus, reassortment profiles of the HK1772 virus with different A(H9N2) viruses might not be extrapolated to infer the reassortment pattern of A(H7N9) viruses of the Yangtze River Delta lineage. Lastly, the A(H9N2) viruses continue to evolve, a recent study showed that G57-lineage viruses isolated since 2015 have contributed to the 5^th^ wave of H7N9 epidemic in humans by providing mammalian adaptive mutations in the *PB2* and *PA* genes [29]. The effect of these adaptive changes in A(H9N2) viruses when co-infecting with A(H7N9) viruses remain to be studied.

Overall, we showed that co-infection of A(H7N9) virus and different A(H9N2) viruses may lead to the emergence of novel reassortants. Our results suggest that most of the reassortants exhibited limited onward transmission potential to contact chickens. These findings provide new insights into the potential mechanism by which influenza viruses may acquire genetic diversity through co-infection *in ovo*, *in vivo*, and under sequential transmission conditions.

## Supplementary Material

Reassortment_supplemental-v13.docx

## Data Availability

Sequences of Sanger sequencing validated in the study were deposited to the public database GISAID under the following accession numbers: HK1772, GISAID accession # EPI_ISL_4882548; G1, GISAID accession # EPI_ISL_3144464; YU335, GISAID accession # EPI_ISL_3144467; HJ, GISAID accession # EPI_ISL_3144489; BJ16, GISAID accession # EPI_ISL_3144854. NGS samples have been deposited in the Sequence Read Archive of the NCBI under project accession number PRJNA768344 and PRJNA769384.
