# Supplementary material for: Limited onward transmission potential of reassortment genotypes from chickens co-infected with H9N2 and H7N9 avian influenza viruses
Source: Emerg Microbes Infect. 2021 Nov 3;10(1):2030–41. doi: 10.1080/22221751.2021.1996209 (PMC8567909; doi:10.1080/22221751.2021.1996209)
Supplement: Reassortment_supplemental-v13.docx [file TEMI_A_1996209_SM4460.docx]

# Supplemental materials

**sFigure 1**. Growth kinetics of H7N9 and H9N2 viruses *in vitro* and *in ovo*. (a) 10-day-old eggs were infected with 10^4^ or 10^6^ plaque-forming units (PFU) of each virus, in triplicate. Allantoic fluid was harvested at the indicated time points, and viral titres were determined as Log_10_(PFU/mL). The means ± standard deviations (SD) of the results are shown. (b) Virus titer changes were calculated by using log_10_(peak virus titer)/ log_10_(start virus titer).A two-tailed Mann-Whitney test was performed to compare the change of virus titer of four avian influenza A(H9N2) viruses in eggs. P value is shown in the figure. (c) Plaque morphology of MDCK cells. MDCK cells seeded in the same 6-well plate were infected with each virus and incubated in infection medium containing 1% agarose for 72 h. The plates were fixed and stained with 0.3% crystal violet. Representative pictures from three independent experiments are shown.


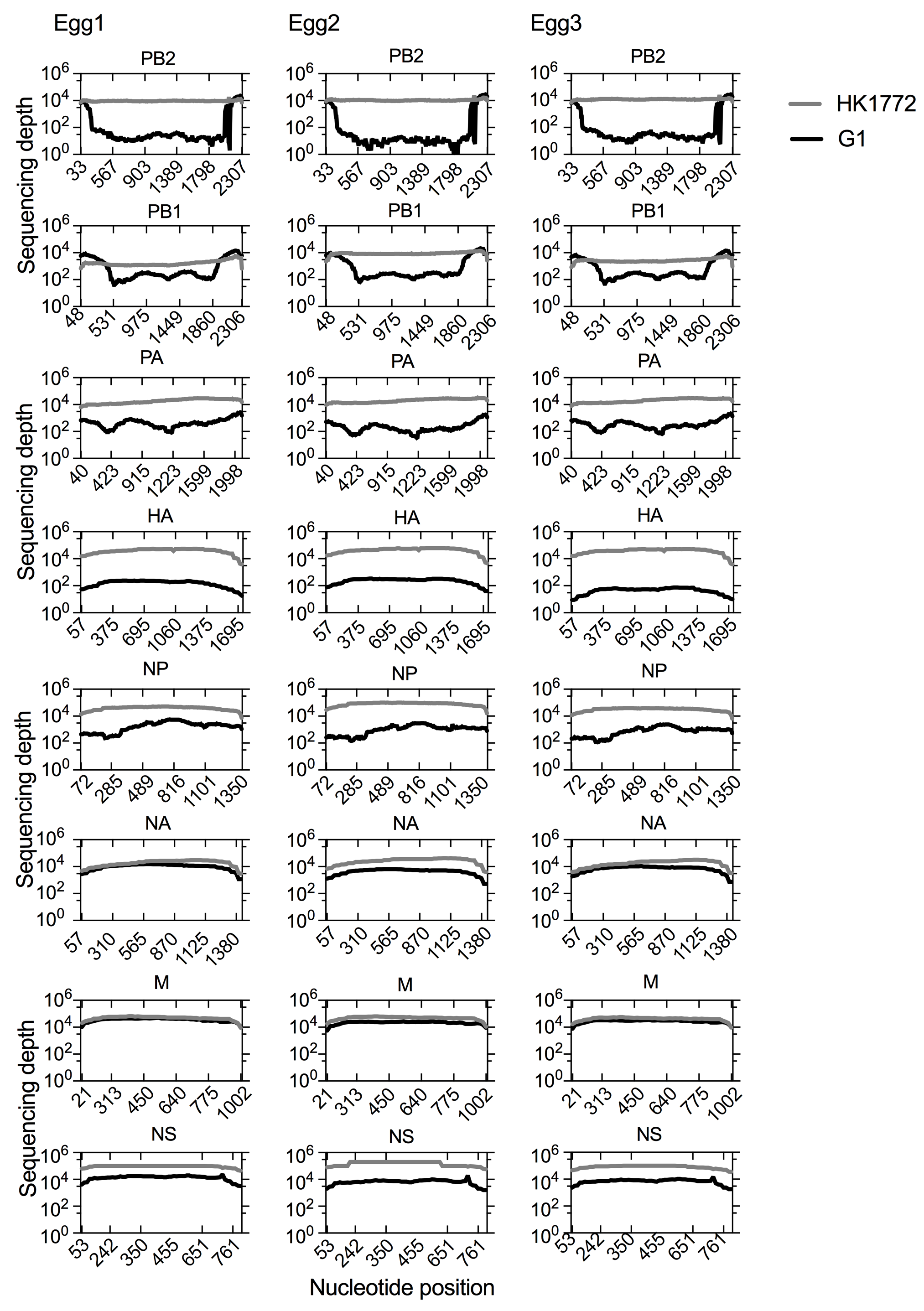


**sFigure 2.** Raw sequencing depth of all segments for eggs co-infected with the A(H7N9) HK1772 virus and the A(H9N2) G1 virus. Three 10-day old embryonated chicken eggs were infected with a mixture of the A(H7N9) virus and the G1 virus at a 1:1 ratio with 10^4^ PFU of each virus in a volume of 0.1 mL.The sequenced population mainly consist of the A(H7N9) strain (gray) in comparison to the A(H9N2) virus (black). The sequencing depth are shown on the y-axis, and each nucleotide position is represented on the x-axis.


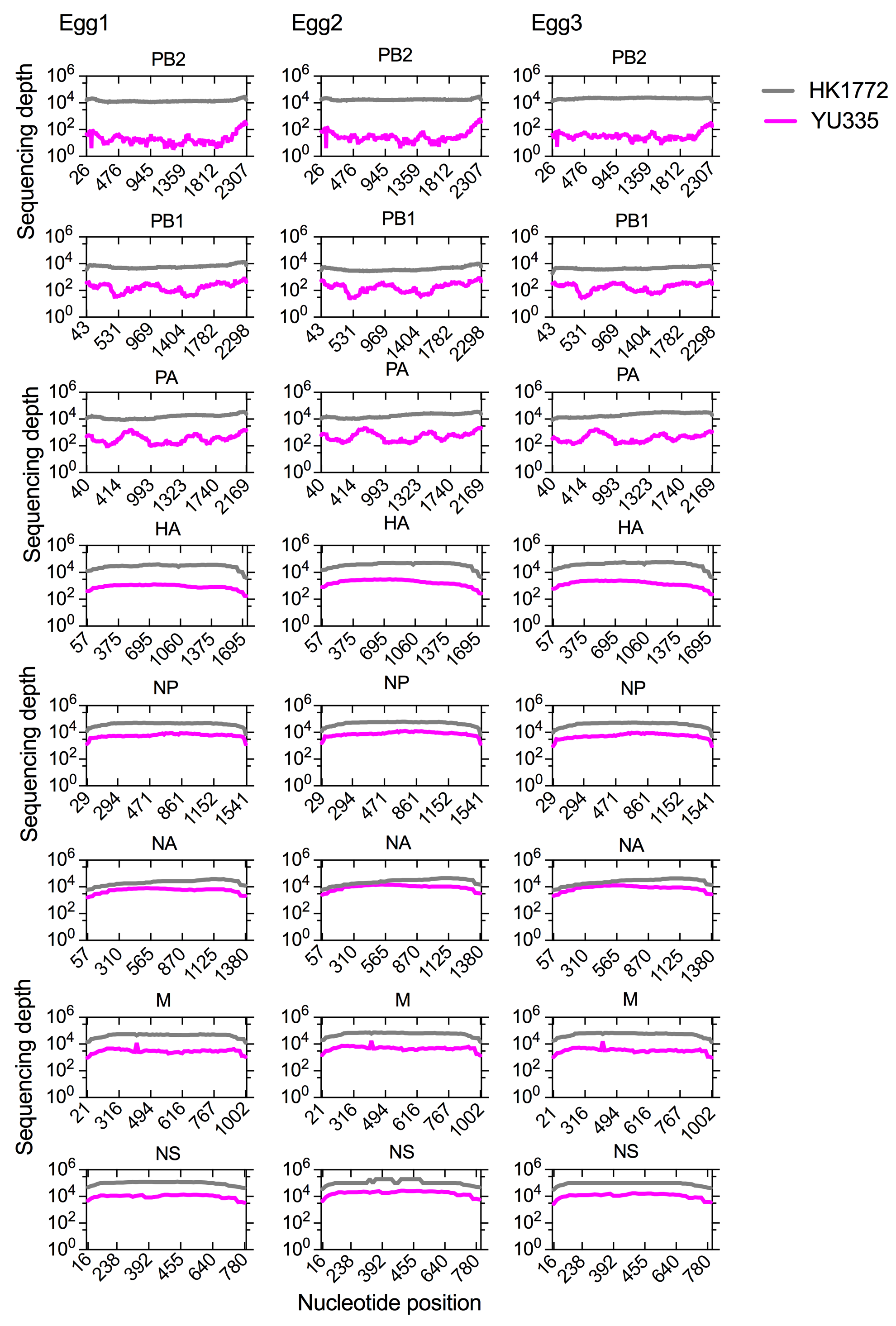


**sFigure 3**. Raw sequencing depth of all segments for eggs co-infected with the A(H7N9) HK1772 virus and the A(H9N2) YU335 virus. Three 10-day old embryonated chicken eggs were infected with a mixture of the A(H7N9) virus and the YU335 virus at a 1:1 ratio with 10^4^ PFU of each virus in a volume of 0.1 mL. in triplicate. The sequenced population mainly consist of the A(H7N9) strain (gray) in comparison to the A(H9N2) virus (pink). The sequencing depth are shown on the y-axis, and each nucleotide position is represented on the x-axis.


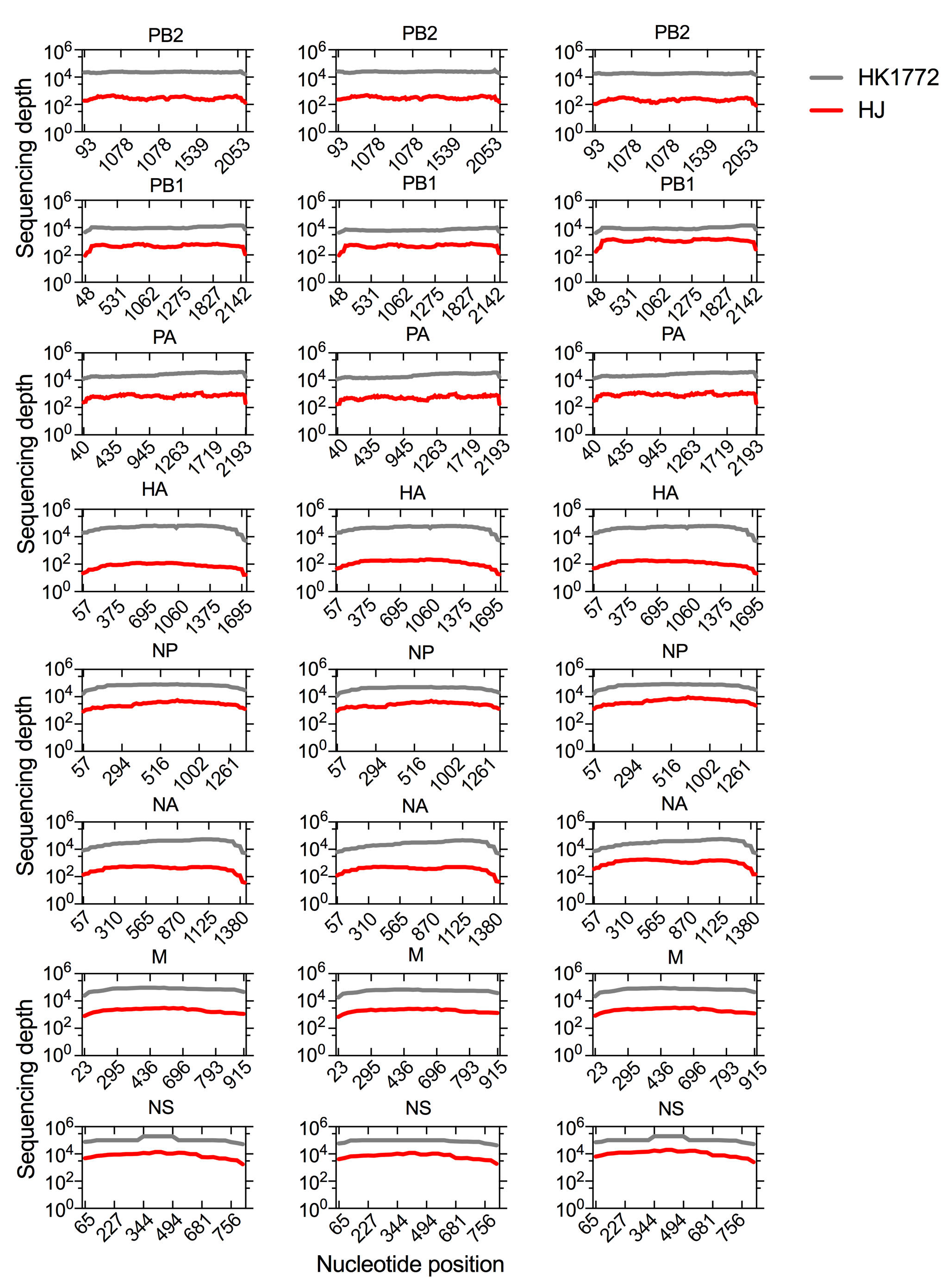


**sFigure 4**. Raw sequencing depth of all segments for eggs co-infected with the A(H7N9) HK1772 virus and the G57 A(H9N2) HJ virus. Three 10-day old embryonated chicken eggs were infected with a mixture of the A(H7N9) virus and the HJ virus at a 1:1 ratio with 10^4^ PFU of each virus in a volume of 0.1 mL. The sequenced population mainly consist of the A(H7N9) strain (gray) in comparison to the A(H9N2) virus (red). The sequencing depth are shown on the y-axis, and each nucleotide position is represented on the x-axis.


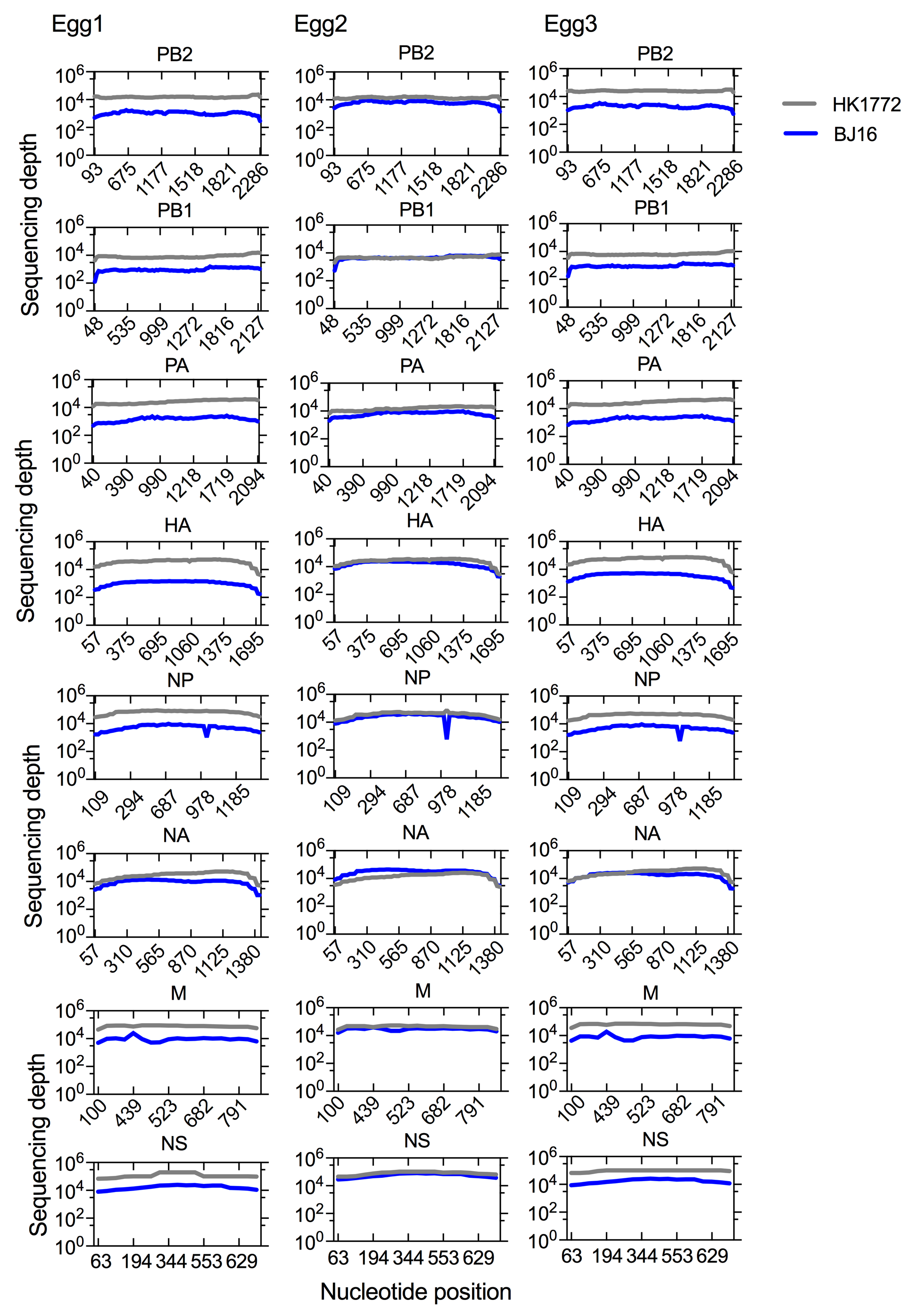


**sFigure 5.** Raw sequencing depth of all segments for eggs co-infected with the A(H7N9) HK1772 virus and the A(H9N2) BJ16 virus. Three 10-day old embryonated chicken eggs were infected with a mixture of the A(H7N9) virus and the BJ16 virus at a 1:1 ratio with 10^4^ PFU of each virus in a volume of 0.1 mL. The sequenced population mainly consist of the A(H7N9) strain (gray) in comparison to the A(H9N2) virus (blue). The sequencing depth are shown on the y-axis, and each nucleotide position is represented on the x-axis.


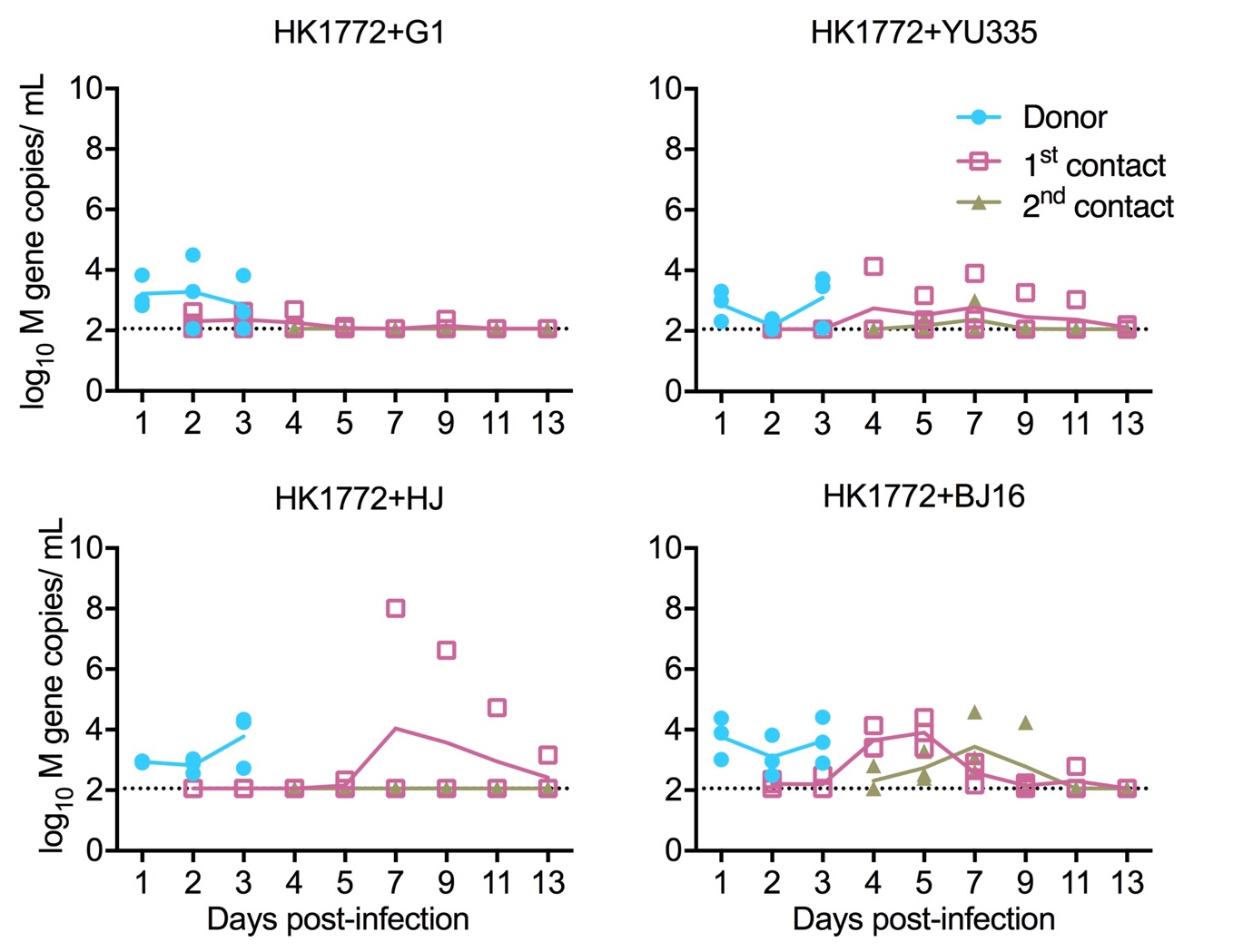


**sFigure 6.** Viral loads detected in cloacal swabs collected from donor and recipient chickens. The lines represent the average number of viral M gene copies in three chickens. The dots indicate the viral load of individual chickens.

**sTable 1.** Primers and probes differentiating eight gene segments of H7N9 and H9N2 viruses

| Segment | Primer name | Sequences (5'-3') |
| --- | --- | --- |
| HA | H7-Forward | AGAAATGAAATGGCTCCTGTCAA |
|  | H7-Reverse | GGCTTTYTCTTGTATTTTTATATGAYTTAG |
|  | H7-Probe | 5HEX-AGATAATGC/ZEN/TGCATTCCCGCAGATG-3IABkFQ |
|  | H9-Forward | CAAGCTGGAATCTGARGGAACTTACA |
|  | H9-Reverse | GCATCTGCAAGATCCATTGGACAT |
|  | H9-Probe | 56-FAM-CCCAGA ACA RGA AGG CAG CAA ACC CCA TTG-BHQ1 |
| NA | N2-forward | TGAGTTRGGTGTCCCGTTTCATTT |
|  | N2-Reverse | GCAGTAGCATTTYTATCATCCCCAGT |
|  | N2-probe | 56-FAM-TGGGAACCA/ZEN/AACAAGTGTGCATAGCATGGTC-3IABkFQ |
|  | N9-Forward | GGAGTGGTTACAGTGGATCTTT |
|  | N9-Reverse | CTTTATCCTCCTTGGGTCTTCC |
|  | N9-Probe | 5HEX-AAACACGCT/ZEN/CGATAGCAGTCCC-3IABkFQ |
| PB2 | PB2-HRM915F* | CAGGCTGTRGATATATGCAA |
|  | PB2-HRM1411R* | TGCTGGGGGTCATGTCAGGCAATAT |
|  | PB2-HRM960F** | TTCATCCTTCAGCTTTGGAGG |
|  | PB2-HRM1411R** | TGCTGGGGGTCATGTCAGGCAATAT |
| PB1 | PB1-HRM99F | AACAGGAACAGGATACACCA |
|  | PB1-HRM293R | GATTCTTCAAGGAAAGCCATT |
| PA | PA-HRM271F | ACAATGGCCTGGACAGTGGT |
|  | PA-HRM479R | TCTTCTCCAGTGAATGAGAA |
| NP | NP-HRM798F | AACCCTGGGAATGCTGAAAT |
|  | NP-HRM942R | CTTCTCTCTCAAAGTCATAT |
| M | M-HRM722F | CTTATTGAAAATTTGCAGGC |
|  | M-HRM900R | GCCCTCTTTTCAAACCGTATTT |
| NS | NS-HRM513F | GCGAAATCTCACCATTACCT |
|  | NS-HRM798R | ACTTCAAGCAATAGTTGTAAGGCTTGC |

* The pair of primers was used to differentiate PB2 of H9N2 G1, HJ, or BJ16 viruses from PB2 of HK1772.

** The pair of primers was used to differentiate PB2 of H9N2 G1, YU335, or BJ16 viruses from PB2 of HK1772.

**sTable 2.** Haemagglutination inhibition (HI) titres against H7N9 virus and H9N2 viruses*

| Group | HK1772+G1 | | |  | HK1772+HJ | | |  | HK1772+YU335 | | |  | HK1772+BJ16 | | |
| --- | --- | --- | --- | --- | --- | --- | --- | --- | --- | --- | --- | --- | --- | --- | --- |
|  | Chicken # | HK1772 | G1 |  | Chicken # | HK1772 | HJ |  | Chicken # | HK1772 | YU335 |  | Chicken # | HK1772 | BJ16 |
| 1^s^ contact | 4 | 64 | <2 |  | 56 | <2 | <2 |  | 17 | <2 | <2 |  | 57 | <2 | 1024 |
|  | 8 | 256 | <2 |  | 63 | <2 | <2 |  | 14 | <2 | 1024 |  | 71 | <2 | 1024 |
|  | 11 | 32 | <2 |  | 66 | 64 | <2 |  | 22 | <2 | 1024 |  | 74 | <2 | 512 |
| 2^nd^ contact | 5 | <2 | <2 |  | 58 | <2 | <2 |  | 42 | <2 | <2 |  | 69 | <2 | 1024 |
|  | 9 | <2 | <2 |  | 64 | <2 | <2 |  | 19 | <2 | 128 |  | 72 | <2 | 1024 |
|  | 12 | <2 | <2 |  | 68 | <2 | <2 |  | 23 | <2 | <2 |  | 75 | <2 | 512 |

* Sera were harvested from contact chickens at the end of the experiment (17 dpi) for the detection of anti-HA antibody against the parental H7N9 virus and the corresponding parental H9N2 virus using haemagglutination inhibition assay (HI).

**sTable 3.** Fisher's exact test with Bonferroni correction was used to identify the correlation of genetic reassortment in eggs with donor chickens.

| Co-infection groups | Eggs vs. chickens at 1 dpi | Eggs vs. chickens at 3 dpi |
| --- | --- | --- |
|  |  |  |
| HK1772+G1 | <0.001 | <0.001 |
| HK1772+YU335 | <0.001 | <0.001 |
| HK1772+HJ | <0.001 | <0.001 |
| HK1772+BJ16 | <0.001 | <0.001 |
